# Laser Soldering with a Biosolder for Oral Mucosa Wound Closure in an Experiment

**DOI:** 10.17691/stm2025.17.4.03

**Published:** 2025-08-29

**Authors:** E.A. Morozova, E.A. Sorokina, A.Y. Gerasimenko, D.I. Ryabkin, V.V. Suchkova, A.A. Timakova, A.L. Fayzullin, S.V. Tarasenko

**Affiliations:** MD, DSc, Associate Professor, Professor, Department of Propaedeutics of Dental Diseases, Medical Institute; Peoples’ Friendship University of Russia named after Patrice Lumumba, 6 Miklukho-Maklaya St., Moscow, 117198, Russia; PhD Student, Department of Surgical Dentistry, E.V. Borovsky Institute of Dentistry; I.M. Sechenov First Moscow State Medical University (Sechenov University), 8/2 Trubetskaya St., Moscow, 119991, Russia; DSc, Associate Professor, Head of the Laboratory of Biomedical Nanotechnology, Institute of Bionic Technologies and Engineering; I.M. Sechenov First Moscow State Medical University (Sechenov University), 8/2 Trubetskaya St., Moscow, 119991, Russia; Head of the Biomedical Nanotechnology Laboratory, Institute of Biomedical Systems; National Research University of Electronic Technology (MIET), 1 Shokin Square, Moscow, Zelenograd, 124498, Russia; PhD, Assistant, Institute of Bionic Technologies and Engineering; I.M. Sechenov First Moscow State Medical University (Sechenov University), 8/2 Trubetskaya St., Moscow, 119991, Russia; Associate Professor, Institute of Biomedical Systems; National Research University of Electronic Technology (MIET), 1 Shokin Square, Moscow, Zelenograd, 124498, Russia; Junior Researcher, Laboratory of Biomedical Nanotechnology, Institute of Bionic Technologies and Engineering; I.M. Sechenov First Moscow State Medical University (Sechenov University), 8/2 Trubetskaya St., Moscow, 119991, Russia; Assistant, Institute of Biomedical Systems; National Research University of Electronic Technology (MIET), 1 Shokin Square, Moscow, Zelenograd, 124498, Russia; Junior Researcher, Laboratory of Digital Microscopic Analysis, Institute for Regenerative Medicine; I.M. Sechenov First Moscow State Medical University (Sechenov University), 8/2 Trubetskaya St., Moscow, 119991, Russia; MD, PhD, Head of the Laboratory of Digital Microscopic Analysis, Institute for Regenerative Medicine; I.M. Sechenov First Moscow State Medical University (Sechenov University), 8/2 Trubetskaya St., Moscow, 119991, Russia; MD, DSc, Professor, Head of the Department of Surgical Dentistry, E.V. Borovsky Institute of Dentistry; I.M. Sechenov First Moscow State Medical University (Sechenov University), 8/2 Trubetskaya St., Moscow, 119991, Russia

**Keywords:** oral mucosa, laser exposure, reparative regeneration, biosolder

## Abstract

**The aim of the study** was to evaluate the efficiency of wound closure of soft tissues using a 970 nm diode laser and a biosolder based on bovine serum albumin and single-wall carbon nanotubes in experimentally modelled oral cavity defects.

**Materials and Methods:**

The experimental study was carried out on 16 chinchilla rabbits. Linear defects of the oral mucosa 1 cm long were modeled. The animals were divided into 2 groups, 8 rabbits in each group. The wounds in the control group animals were sutured with a surgical suture using Prolene 5-0 thread; the experimental group animals were sutured using laser soldering and a biosolder based on bovine serum albumin, indocyanine green, single-wall carbon nanotubes, and type I collagen, followed by putting additional sutures using Prolene 5-0 thread. We used the proprietary laser device with a wavelength of 970 nm with adaptive thermal stabilization of the suture, which enabled to set the heating temperature of the biotissue in the laser suture area with an accuracy of ~1°C preventing thermal necrosis of tissues.

The biological tissues of 24 samples of the rabbit oral mucosa were fixed on days 1, 3, 5, and 10 and examined morphologically and morphometrically.

**Results:**

Inflammatory changes were primarily associated with a response to the suture material; proliferative changes (neoangiogenesis and epithelial regeneration) were related to the proliferation activation of fibroblasts and epithelial cells due to the laser exposure. The use of a biosolder contributed to additional tissue adhesion, which further on accelerated the regeneration process and increased the neoangiogenesis rate and the vascular density per 1 mm^2^.

In the experimental group, the inflammatory reaction was completed by day 5, while in the control group, the residual inflammatory signs persisted in some samples up to day 10. On day 10, the proliferative phase began in the experimental group. An immunohistochemical analysis revealed a statistically significant increase in the number of blood vessels in the experimental group by 70.6% compared to the control (p=0.003).

**Conclusion:**

The use of laser exposure combined with a biosolder promoted tissue adhesion improvement, shortened the inflammatory phase, and accelerated the regeneration providing minimal scarring. The data obtained emphasize the prospects of using the suggested technique for oral mucous wound closure in clinical practice for patients with various dental diseases.

## Introduction

Laser medicine is one of the research priorities in modern dentistry. Laser soldering has a number of advantages compared with traditional methods applied for reconstructing biological tissues using surgical suture materials (e.g., needles and suture). The advantages include wound integrity and sterility, vascular anastomosis, nearly unnoticeable scars on the suture site, and rapid tissue adhesion [1, 2]. It has a direct effect on the treatment quality and the acceleration of reparative processes [3–7]. Researchers emphasize a diode laser to be highly safe, and due to this characteristic, it can be used in dentistry, since it has no damage effect on soft tissues [8].

For the purpose of accelerating reparative processes and better soldering of wound edges, special biosolders are used during a laser-aided surgery to close wound tissues. A biosolder can contain such proteins as albumin, fibrinogen, and collagen. Recently, a new composition of biosolder was suggested based on the biological material of albumin, a filler from single-wall carbon nanotubes and a medical stain, indocyanine green. Such a biosolder provides high tensile strength of a laser suture, has low content of carbon nanotubes, minimizing the energetic load of laser radiation and accelerating the soldering process [9].

Laser tissue soldering is especially efficient in recovering the integrity of small blood vessels, nerve fibers, and spermatic ducts. However, laser devices do not always provide sufficient contraction strength of biological tissues. Accordingly, it is necessary to enhance the laser soldering efficiency of wound tissues (e.g., through the absorption increase of the used laser radiation), it will enable to provide precise heating of the soldered wound to avoid overheating of the surrounding tissues [10–15].

When closing the wound edges including oral surgeries, the following things are of great importance: the suture integrity along with the possibility to prevent inflammation and necrosis, and the esthetic quality of a postoperative scar [1, 2, 4, 5, 16, 17]. Currently, there are no universal technique suggested for soldering wound edges in surgical treatment of patients with dental disorders; a technique should comply with all requirements and have no application restrictions.

**The aim of the study** was to evaluate the efficiency of wound closure of soft tissues using a 970 nm diode laser and a biosolder based on bovine serum albumin and single-wall carbon nanotubes in experimentally modelled oral cavity defects.

## Materials and Methods

### Animal studies

The study was carried out in full accordance with the ethic principles stated by European Convention for the Protection of Vertebrate Animals used for Experimental and Other Scientific Purposes (Convention was passed in Strasburg, March 18, 1986, adopted in Strasburg, June 15, 2006), and approved by the Ethics Committee of I.M. Sechenov First Moscow State Medical University. The animals were managed according to the requirements of GOST P 53434-2009 “Principles of good laboratory practice (GLP)” (December 2, 2009). The animals were fed in accordance with the standards in agreement with an animal species, using no special nutrition (e.g., feed containing supplements for regeneration stimulation).

The study involved 16 chinchilla rabbits divided into two groups — 8 rabbits in each group. Animals in each group was distributed by four time points (2 animals per each point). Linear defects of the maxillary facial surface on either side, 1 cm long, were modeled using scalpel No.15C in all animals. Additionally, in one of two rabbits at each time point, we modeled a defect of the mandible on the right, and an intact mucosal sample was taken from another rabbit. Thus, on each time point we had six samples: five samples from the wound healing area and one sample — from the intact tissue.

The wound edges in both groups (the experimental and the control) were closed under general anesthesia and local anesthesia using injectable solution Articaine Binergia with adrenalin (20 mg + 0.005 mg/ml).

The wounds in the control group animals were sutured with a surgical suture using Prolene 5-0 thread; the experimental group animals were sutured using laser soldering and a biosolder based on bovine serum albumin, indocyanine green, single-wall carbon nanotubes, and type I collagen, followed by putting additional sutures using Prolene 5-0 thread.

In the experimental group, the biosolder was placed into the wound using a crescent burnisher equally along the edges; in case of applying excessive biosolder, it was removed using a sterile gauze swab by soaking it on the wound surface followed by the wound being exposed to laser radiation by sliding the laser column from the incision edge throughout its length. The laser suture, 1 cm long, was being formed for 15 s. Three interrupted stitches were put on a laser suture to strengthen it. We used the laser device with a wavelength of 970 nm with adaptive thermal stabilization of the suture developed in the Biomedical Nanotechnology Laboratory, Institute of Biomedical Systems, Moscow National Research University of Electronic Technology. The device enabled to set the heating temperature of the biotissue in the laser suture area with an accuracy of ~1°C preventing thermal necrosis of the tissue. The optic fiber diameter was 600 μm; the laser spot diameter — 2 mm. The average temperature in the laser soldering area was 40°; the laser radiation power ranged from 1.1 to 2.5 W.

The laboratory animals of both groups were sacrificed on postoperative days 1, 3, 5, and 10 by injecting them with an excessive dose of Zoletil. The oral mucosa samples of the rabbits were sent for histology and immunohistochemistry analyses.

### Histological study

48 samples from 16 animals were subjected to the histological analysis; among them 20 samples had oral mucosal defects after suturing, and 20 samples — after using laser soldering. The rest 8 samples were intact oral mucosa. Thus, in each group there were 5 samples subjected to the histological analysis for each time point.

The tissues were fixed in 10% neutral buffered formalin and embedded into paraffin blocks in strict orientation to obtain the sections perpendicular to the mucosa surface. The sections 3–4 μm thick were stained with hematoxylin and eosin and Mallory’s trichrome. The samples were studied by standard optical microscopy using a universal microscope Leica DM4000 B equipped with a video camera Leica DFC7000 T and software LAS V4.8 (Leica Microsystems, Germany).

Each preparation was assessed for inflammatory signs (exudation, infiltration by immune cells, microcirculatory disorders) and regeneration signs (neoangiogenesis, proliferation of fibroblasts) according to the previously published 4-point scale (see the Table) [18].

**Table:** Scoring system of morphological signs in the treatment area [18]

Scores	Exudation	Infiltration by immune cells	Microcirculatory injuries	Neoangiogenesis	Fibroblast proliferation
0	No signs	No signs	No signs	No signs of new vessels forming	No signs
1	Mild edema, in the intercellular space there is a small amount of liquid	Infiltration by singular immune cells (less than 10 cells in 1 field of vision; 400×)	Red blood cell margination (mural) position in the vessel lumen	The beginning of vascular formation: no vascular wall, the endothelium is a thin layer of endothelial cells	Mild hypertrophy and hyperplasia of fibroblasts, their volume increasing by less than 10%
2	Moderate edema, in the intercellular space there is a moderate amount of liquid	Moderate infiltration by immune cells (from 11 to 29 cells in the field /of vision; 400×)	Initial manifestations of red blood cell aggregation and agglutination in the vessel lumen	Continuation of vessel formation: *t. adventitia* is absent, muscular fibers in *t. media* are thin), the endothelium has normal structure	Moderate signs of hypertrophy and hyperplasia of fibroblasts, their volume increasing by 20–30%
3	Pronounced tissue edema, in the intercellular space there is a considerable amount of liquid	High infiltration degree by immune cells (over 30 cells in the field of vision; 400×)	Red blood cell stasis and aggregation in the vessel lumen	The vessels are completely formed: the wall has three-layer structure (*t. adventitia, t. media, t. intima*), the endothelium has normal structure	Prominent signs of hypertrophy and hyperplasia of fibroblasts, their volume increasing by more than 30%

### Immunohistochemistry analysis

The sections 3–4 μm thick were deparaffinized and incubated with 3% hydrogen peroxide for 10 min. Nonspecific staining was prevented using the blocking solution (Cell Marque, USA). The sections were incubated with murine monoclonal primary antibodies against α-smooth muscle actin, or α-SMA (A2547; Merck & Co., Inc., USA; dilution 1:400). The sections were imaged using secondary goat antibodies conjugated with horseradish peroxidase (G-21040; Invitrogen, USA; dilution 1:1000), and diaminobenzidine (DAB) with contrast hematoxylin staining. α-SMA expression in implantation areas was assessed by a semi-quantitative system:

“–” — no expression;

“+” — singular positively stained cells;

“++” — small amount of positively stained cells (less than 19 per 1 field of vision; 400×);

“+++” — considerable amount of positively stained cells (more than 20 per 1 field of vision; 400×).

Vascular density was determined using the microscope Leica DM4000 B T (Leica Microsystems, Germany) at 200× in 5 representative fields of vision.

### Statistical analysis

The experimental findings were statistically analyzed using the software Prism 10.0 for Windows (GraphPad, USA). The differences in morphometrical findings (exudation, infiltration by immune cells, microcirculatory disorders, neoangiogenesis, proliferation of fibroblasts) were assessed using Mann– Whitney test. The normal distribution of the quantitative data (the number of blood vessels calculated per 1 mm^2^) was checked using Shapiro–Wilk’s test. The differences in blood vessel density were determined by t-test. The results were considered significant if p≤0.05. The statistical findings of the scoring morphometric parameters were represented in the form of column graphs with median values and interquartile ranges (Me [Q1; Q3]). The vascular density analysis results were represented as mean values calculated per 1 mm^2^, with the reference to a standard deviation (Me±SD).

## Results

**Figure 1.**
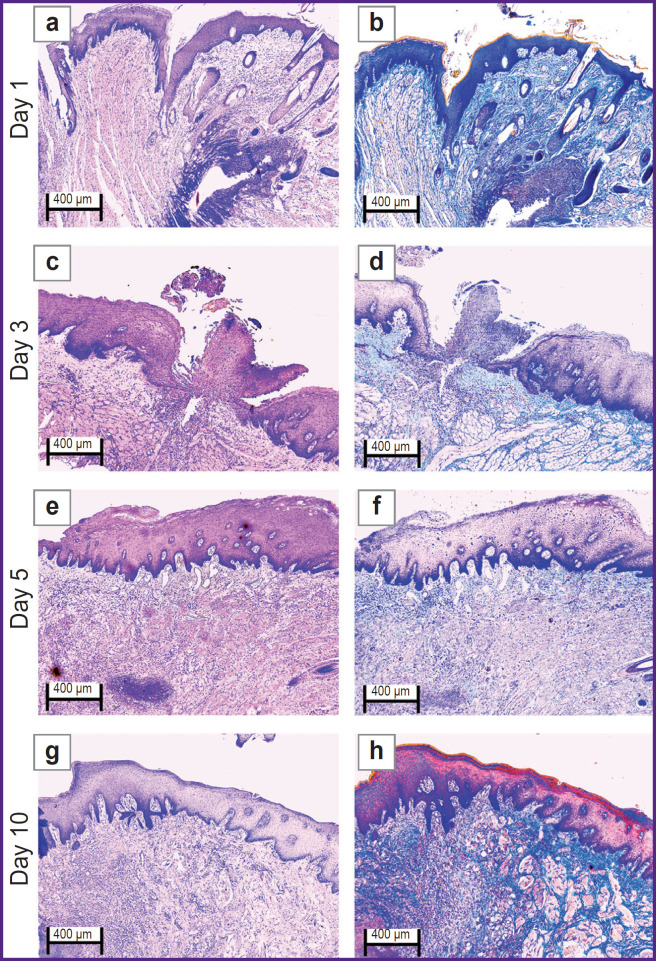
Rabbit oral mucosa microstructure in the control group (the defect is sutured with Prolene 5-0): (a), (c), (e), (g) hematoxylin and eosin staining; (b), (d), (f), (h) Mallory’s staining; 50×

**Figure 2.**
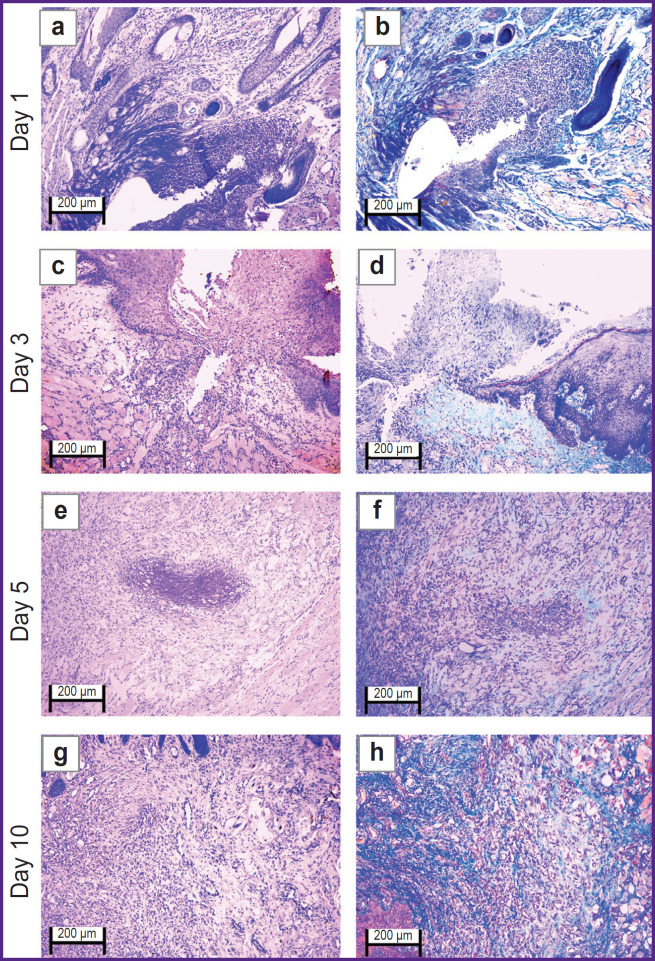
Rabbit oral mucosa microstructure in the control group (the defect is sutured with Prolene 5-0): (a), (c), (e), (g) hematoxylin and eosin staining; (b), (d), (f), (h) Mallory’s staining; 100×

The oral mucosal tissue fragments in the ***control group*** on day 1 (Figure 1 (a), (b) and Figure 2 (a), (b)) were lined with stratified squamous epithelium. Three of five sections under study had a mucosa defect in the synthetic suture area with impaired epithelial lining, surrounded by inflammatory cells (primarily neutrophils and lymphocytes). The submucosa there was slightly edematous and loose, contained dilated full-blood vessels of a capillary type. Mallory’s staining revealed loose bright blue collagen fibers, and paler fibers — in the edema region. The sections also had protein-mucous glands and adipose tissue with no abnormalities.

By day 3 (Figure 1 (c), (d) and Figure 2 (c), (d)) the histological pattern of the inflammatory phase of wound repair had no significant changes; however, among immune cells, macrophages were observed.

On day 5 (Figure 1 (e), (f) and Figure 2 (e), (f)) the oral mucosa was lined with stratified squamous epithelium. One section was found to have the mucosa defect (with the decreased area) in the synthetic suture region surrounded by the moderate number of inflammatory cells (macrophages, lymphocytes, and singular neutrophils). Two sections had the epithelial continuity, but the suture material was surrounded by macrophages, lymphocytes, and singular neutrophils. The lamina propria was edematous and loose, contained full-blood vessels of a capillary type; the edema was more localized. Mallory’s staining made loose collagen fibers of the lamina propria bright blue; in some edema regions they were pale blue with lilac inclusions (microsomatosis). Compared to days 1 and 3, the inflammatory edema became smaller and more localized; there were few neutrophils (the acute phase cells).

On day 10 (Figure 1 (g), (h) and Figure 2 (g), (h)) the oral mucosa was lined with regenerative stratified squamous epithelium with the signs of the basal layer proliferation. One of five sections was found to have the epithelizing mucosa defect; no suture fragments were revealed. The submucosa contained moderate quantity of lymphocytes and the network of capillary-type vessels. Mallory’s staining revealed loose bright blue collagen fibers. Compared to days 1, 3, and 5, there were no acute inflammatory signs (neutrophils); the epithelium had the regenerative signs; there was no edema.

**Figure 3.**
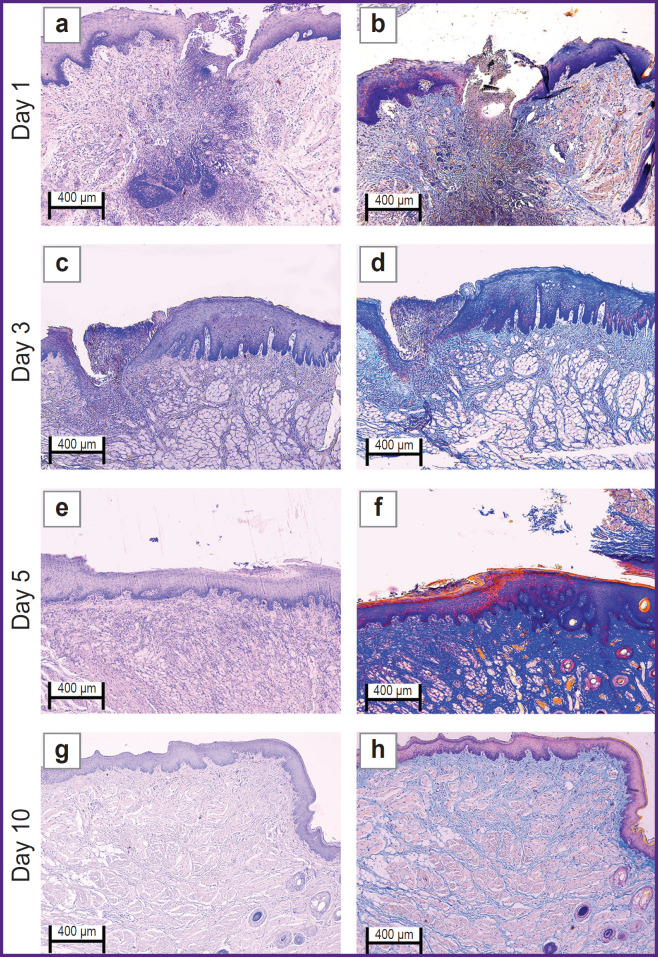
Rabbit oral mucosa microstructure in the experimental group (laser soldering of the wound using a biosolder and an additional suture with Prolene 5-0) (a), (c), (e), (g) hematoxylin and eosin staining; (b), (d), (f), (h) Mallory’s staining; 50×

**Figure 4.**
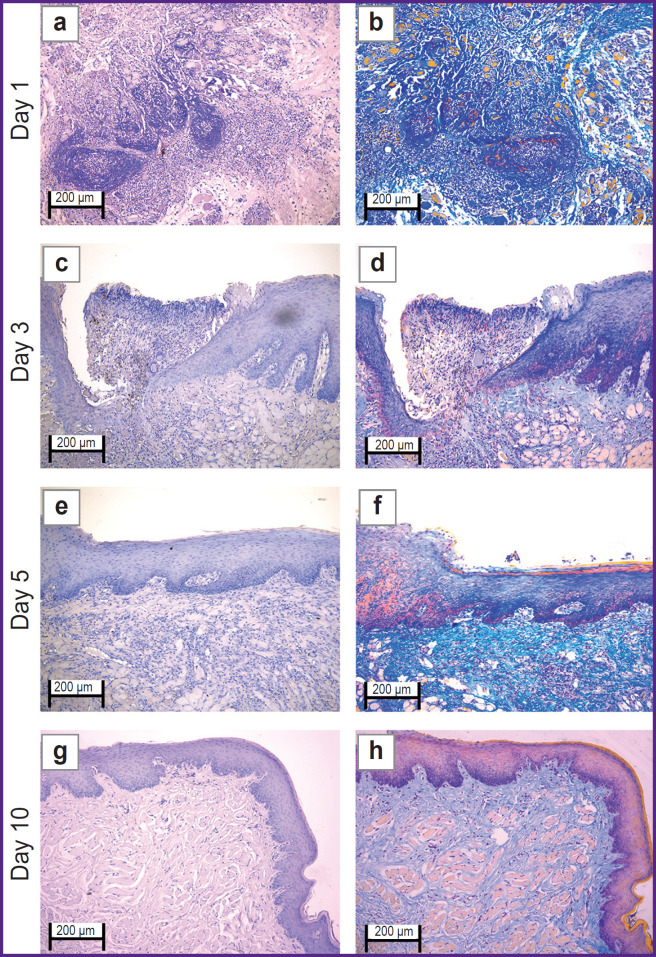
Rabbit oral mucosa microstructure in the experimental group (laser soldering of the wound using a biosolder and an additional suture with Prolene 5-0) (a), (c), (e), (g) hematoxylin and eosin staining; (b), (d), (f), (h) Mallory’s staining; 100×

In the ***experimental group*** on day 1 (Figure 3 (a), (b) and Figure 4 (a), (b)) the oral mucosa was lined with stratified squamous epithelium. Two of five sections had the mucosa defect in the synthetic suture area with impaired epithelial lining and overlapping fibrin surrounded by inflammatory cells (primarily, neutrophils and lymphocytes). The submucosa was edematous, loose, with fibrinoid necrotic patches, and contained dilated full-blood vessels of a capillary type. Additionally, the sections had protein-mucous glands and adipose tissue with no abnormalities. Mallory’s staining revealed bright blue collagen fibers of the lamina propria to have no dystrophic changes; and in the fibrinoid necrotic patches there was the homogenized fibers of pale blue color. No significant differences from the control sample were found.

By day 3 (Figure 3 (c), (d) and Figure 4 (c), (d)) the inflammatory phase of the wound repair became less marked due to a decreased number of neutrophils and the absence of edema. There was observed active epithelization of the mucosa defects and the epithelial growth under the necrotic detritus.

On day 5 (Figure 3 (e), (f) and Figure 4 (e), (f)) not all sections were found to have mucosa defects and suture material. In the former alteration region in the lamina propria, cell-dense connective tissue was observed. Mallory’s staining revealed loose bright blue collagen fibers, which were more intensely stained in the regeneration region. Compared to day 3, there were neither defects nor acute inflammatory signs. In the control, on day 5, the edema, myxomatosis, neutrophil infiltration were still observed; so, the sections differed significantly compared to the samples of two other groups.

On day 10 (Figure 3 (g), (h) and Figure 4 (g), (h)) the mucosa was lined with stratified squamous epithelium with the proliferation signs of the basal layer. Beneath the epithelium the lamina propria was observed — compactly packed loose connective tissue containing moderate quantity of lymphocytes and the network of capillary-type vessels (neoangiogenesis). Mallory’s staining revealed loose bright blue collagen fibers. In contrast to the controls, in this time point, the post-laser soldering wound had no inflammatory signs, the wound repair was completed.

**Figure. 5:**
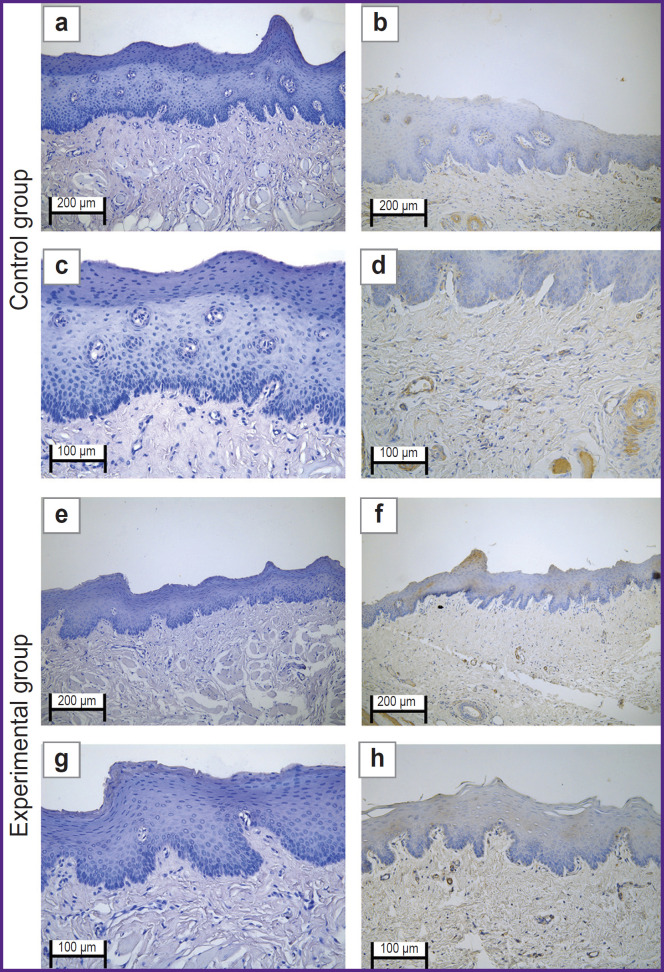
Vascularization of oral mucosa defects after wound edge closure without (a)–(d) and with laser soldering (a), (c), (e), (g) hematoxylin and eosin staining; (b), (d), (f), (h) imunohistochemical reaction with the antibodies against alpha-smooth muscle actin (α-SMA), hematoxylin contrast; (a), (b), (e), (f) 100×; (c), (d), (g), (h) 200×

In the immunohistochemical study with the antibodies to α-SMA in the ***control group*** on day 10, there was poor expression in the vascular muscular wall (+). The vascular density was 320 per 1 mm^2^ of the slide (Figure 5 (a)–(d)).

The immunohistochemical study with the antibodies to α-SMA in the ***experimental group*** revealed moderate expression in fibroblasts and in smooth muscle cells in the vascular wall (++). The vascular density was 550 per 1 mm^2^ of the slide (Figure 5 (e)–(h)).

**Figure 6.**
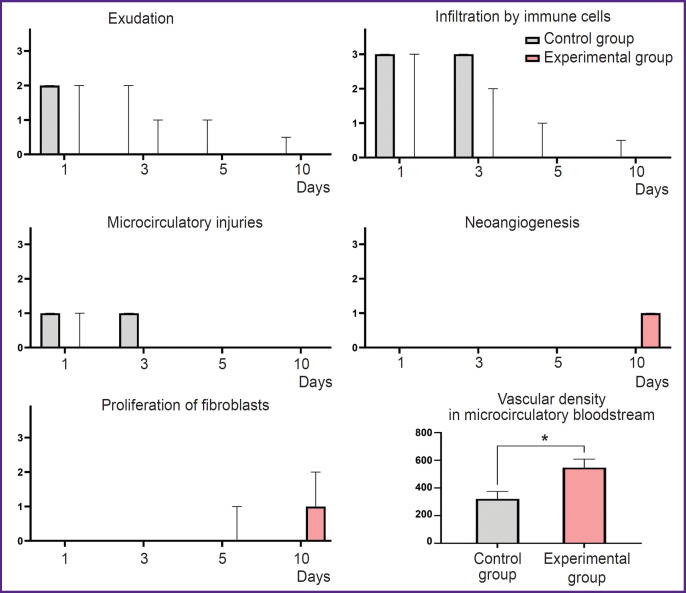
Statistical analysis of the morphological signs of inflammation, regeneration and density of the vessels of the microcirculatory bloodstream (the number of blood vessels per 1 mm^2^) in the control (suture) and experimental (suture and laser soldering) groups Me [Q1; Q3] — for morphometric scoring (exudation, infiltration by immune cells, microcirculatory injuries, neoangiogenesis, proliferation of fibroblasts) and Me±SD — for the density of vessels of microcirculatory bloodstream. * p≤0.001

The morphometric analysis showed the use of laser soldering contributed to the promoted completion of the inflammatory phase of a wound process. It was manifested by a total absence of the signs of exudation, infiltration by immune cells and impaired microcirculation. In the experimental group the inflammatory reaction was completed by day 5, while in the control group the residual inflammatory signs were persisting in some samples up to 10 days. On day 10 in the experimental group the proliferative phase started: in some samples we could observe the active proliferation of fibroblasts and the formation of the regions with loose connective tissue with increased cellularity. However, an immunohistochemistry assay revealed the significant increase by (70.6%) of the number of blood vessels in the experimental group compared to the control (p=0.003) (Figure 6).

## Discussion

The suggested technique of laser soldering of oral mucous soft tissues includes connecting the postoperative wound edges using a biosolder and a laser apparatus based on a diode laser with a wavelength of 970 nm with an adaptive suture thermal stabilization. The proprietary laser device was equipped with the temperature feedback system, which was operated using a bolometric infrared matrix sensor. The sensor determines the hottest point in the laser soldering site of the biological tissues, and regulates the currency on a laser diode. The target temperature of laser soldering was set with accuracy up to 1°C preventing tissue overheating and necrosis. To improve the strength of welded joints we used a biosolder to increase laser radiation absorption and prevent thermal necrosis of tissues. A biosolder was the aqueous dispersion of bovine serum albumin, indocyanine green and single-wall carbon nanotubes. A biosolder exposed to the laser radiation made the disperse liquid transform into a nanocomposite, which was a framework structure of carbon nanotubes in a biopolymer matrix providing the soldering of the wound edges and the formation of a strong laser welded suture. The oral mucosa edges are characterized by high liquid concentration (saliva); therefore, the biosolder composition was added by a protein — type I collagen — increasing its viscosity till gel state; it prevented the biosolder from leaking out of the wound.

The findings correlate with the data of our previous study devoted to laser soldering of skin. We found [9] observing the soldering rules and the task precision to result in less pronounced inflammatory reaction and less marked scarring. Aneurisms are less common in laser soldering of vessels. Carbon nanotubes in the biosolder composition promote the increase of laser suture tensile strength. Additionally, the operation duration and labor consumption decrease.

According to histological examination, in the experimental group, when soldering the wound edges (laser suture; the biosolder consisting of bovine serum albumin, indocyanine green, single-wall carbon nanotubes, and type I collagen; Prolene 5-0) the operation zone on day 1 was determined in the form of the irregular-shaped fibrinoid necrotic focus, the mild inflammation was due to the response to a suture thread. In the control group (a surgical suture using Prolene 5-0 thread) inflammatory manifestations were expressed in a greater degree (compared to the experimental group). On day 3 the inflammatory manifestations in the experimental group were minimal, while those in control group were persisting: epithelialization was impaired, there were inflammatory cells (primarily neutrophils, lymphocytes, and macrophages). Proliferative (neoangiogenesis and regeneration of the epithelium) changes in the experimental group were to a greater degree due to active proliferation of fibroblasts following the laser radiation exposure. The alterations were maximally expressed on day 10. According to the immunohistochemistry assay findings, the use of laser radiation combined with a biosolder and additional suturing with Prolene 5-0 thread to close the oral mucosa wound edges contributed to effective tissue soldering that accelerated the regeneration process and neoangiogenesis. It is the significant advantage of the laser tissue soldering technique.

## Conclusion

The present *in vivo* study demonstrated the efficiency of closing oral mucosa wound edges using laser radiation and a biosolder based on bovine serum albumin, indocyanine green, single-wall carbon nanotubes and type I collagen. The suggested method enables to avoid tissue overtension and injury, promotes additional tissue adhesion. Moreover, laser soldering with a biosolder shortens an inflammatory phase and improves the regenerative potential of postoperative tissues.
